# P-1901. Statin Use and Severe/Critical/Fatal Outcomes in Individuals with COVID-19

**DOI:** 10.1093/ofid/ofae631.2062

**Published:** 2025-01-29

**Authors:** Adeel A Butt, Peng Yan, Obaid Shaikh

**Affiliations:** Weill Cornell Medicine, Doha, Ad Dawhah, Qatar; VA Pittsburgh Healthcare System, Pittsburgh, Pennsylvania; VA Pittsburgh Healthcare System, Pittsburgh, Pennsylvania

## Abstract

**Background:**

There are conflicting results regarding the effects of statins upon outcomes in individuals with COVID-19. Several studies demonstrated a beneficial effect on survival, with others failing to demonstrate a beneficial effect, or even a higher risk of mortality, need for mechanical ventilation, and intensive care unit admissions among those who received statins.

Statins in COVID_Fig 1

**Figure:**
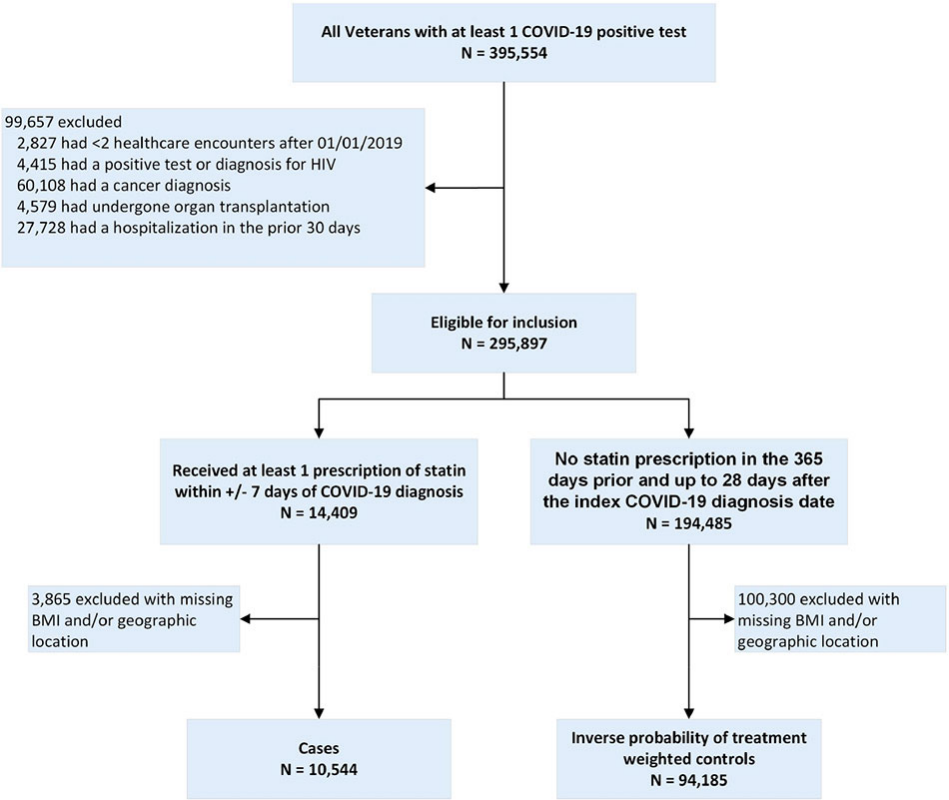

**Methods:**

We identified all individuals in the VA COVID-19 database with confirmed COVID-19. We excluded those with HIV, organ transplantation, cancer diagnosis, and any hospitalization within preceding 30 days. Among those, we created a pool of potential cases who had received a statin within +/- 7 days, and controls with no statins in the previous 365 days and up to 28 days after the index positive test date. Using an inverse probability of treatment weights (IPTW) based approach, we determined the rates of moderate/severe/critical disease within 28 days of the index COVID-19 diagnosis among statin-recipients and controls. Absolute risk differences (ARD) and Kaplan-Meier curves were generated for cases and controls.

Statins in COVID_Fig 2

**Figure:**
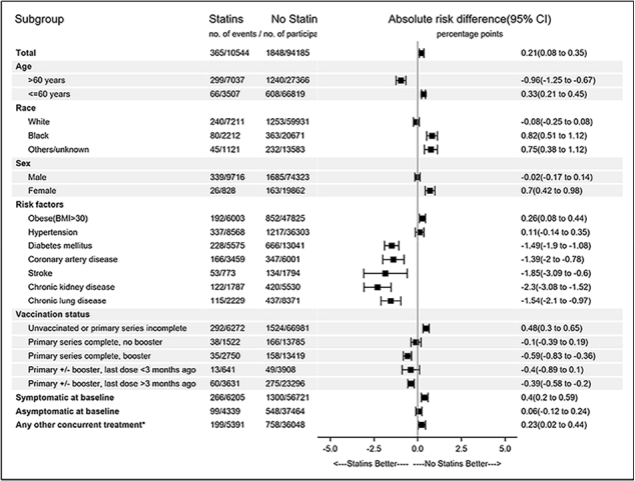

**Results:**

In IPTW-weighted matched analysis including 10,544 statin-recipients and 94,185 controls, statin-recipients were more likely to have an unfavorable outcome compared with controls (ARD 0.21 (95% CI 0.08,0.35) overall. In subgroup analyses, statin-recipients >60 years old and those with diabetes, cardiovascular disease, or chronic lung or kidney disease had more favorable outcomes compared with controls.

**Conclusion:**

Individuals with COVID-19 who received statins were more likely to have poorer outcomes compared with controls not receiving statins. Subgroup analyses demonstrating a benefit among those at high risk of cardiovascular outcomes may be reflection of the beneficial effects of statins on the underlying comorbidities rather than a direct benefit against COVID-19.

**Disclosures:**

Adeel A. Butt, MD, MS, Gilead Sciences: Grant/Research Support

